# Epidemiologic features of overseas imported malaria in the People's Republic of China

**DOI:** 10.1186/s12936-016-1188-7

**Published:** 2016-03-05

**Authors:** Zhongjie Li, Qian Zhang, Canjun Zheng, Sheng Zhou, Junling Sun, Zike Zhang, Qibin Geng, Honglong Zhang, Liping Wang, Shengjie Lai, Wenbiao Hu, Archie C. A. Clements, Xiao-Nong Zhou, Weizhong Yang

**Affiliations:** Division of Infectious Diseases, Key Laboratory of Surveillance and Early-warning on Infectious Disease, Chinese Center for Disease Control and Prevention, 155 Changbai Road, Changping, 102206 Beijing China; Center of Clinical Laboratory, the First Affiliated Hospital, College of Medicine, Zhejiang University, Hangzhou, China; State Key Laboratory of Virology and College of Life Sciences, Wuhan University, Wuhan, China; Department of Geography and Environment, University of Southampton, Southampton, UK; School of Public Health and Social Work, Queensland University of Technology, Brisbane, Australia; Research School of Population Health, College of Medicine, Biology and Environment, The Australian National University, Canberra, Australia; National Institute of Parasitic Diseases, Chinese Center for Disease Control and Prevention, Key Laboratory of Parasite and Vector Biology, MOH, WHO Collaborating Centre for Tropic Diseases, National Center for International Research on Tropical Diseases, 207 Rui Jin Er Road, Shanghai, 200025 People’s Republic of China; Key Laboratory of Surveillance and Early-warning on Infectious Disease, Chinese Center for Disease Control and Prevention, Beijing, China

**Keywords:** Malaria, Imported, Epidemiology, China

## Abstract

**Background:**

With the dramatic increase in international travel among Chinese people, the risk of malaria importation from malaria-endemic regions threatens the achievement of the malaria elimination goal of China.

**Methods:**

Epidemiological investigations of all imported malaria cases were conducted in nine provinces of China from 1 Nov, 2013 to 30 Oct, 2014. *Plasmodium* species, spatiotemporal distribution, clinical severity, preventive measures and infection history of the imported malaria cases were analysed using descriptive statistics.

**Results:**

A total of 1420 imported malaria cases were recorded during the study period, with *P. falciparum* (723 cases, 50.9 %) and *P. vivax* (629 cases, 44.3 %) being the two predominant species. Among them, 81.8 % of cases were in Chinese overseas labourers. The imported cases returned from 41 countries, mainly located in Africa (58.9 %) and Southeast Asia (39.4 %). About a quarter (25.5 %, 279/1094) of counties in the nine study provinces were affected by imported malaria cases. There were 112 cases (7.9 %) developing complicated malaria, including 12 deaths (case fatality rate: 0.8 %). Only 27.8 % of the imported cases had taken prophylactic anti-malarial drugs. While staying abroad, 27.7 % of the cases had experienced two or more episodes of malaria infection. The awareness of clinical manifestations and the capacity for malaria diagnosis were weak in private clinics and primary healthcare facilities.

**Conclusions:**

Imported malaria infections among Chinese labourers, returned from various countries, poses an increasing challenge to the malaria elimination programme in China. The risk of potential re-introduction of malaria into inland malaria-free areas of China should be urgently addressed.

## Background

Malaria, transmitted via the bite of infected *Anopheles* mosquitoes, is one of the most important parasitic diseases to affect mankind with a heavy burden of disease [1]. Globally an estimated 3.3 billion people are at risk of being infected with malaria and developing disease. In 2013, 198 million cases of malaria occurred, leading to 584,000 deaths [2]. Due to joint efforts made by the international community, the global burden of malaria has decreased substantially during recent decades [3], with a 47 % decline in malaria mortality rates globally, averting an estimated 4.3 million deaths between 2001 and 2013 [4]. In 2015, the World Health Organization (WHO) set an ambitious new target of reducing the global malaria burden by 90 % by 2030, and encouraged nation members to fulfill the goal of malaria elimination [4].

Malaria is a mandatory notifiable infectious disease in the People's Republic of China, with each case required to be reported through the National Infectious Disease Reporting Information System [5]. Historically, malaria has been the most prevalent infectious disease in P.R.China, accounting for more than 24 million cases during the early 1970s [6]. Long-term implementation of anti-malaria campaigns in areas with high transmission of malaria in P.R.China, including strengthening surveillance systems, improving access to treatment, preventive anti-malarial administration for high-risk groups, environmental improvement, vector control, and social mobilization, has resulted in an unprecedented decrease in number of malaria-endemic areas in mainland China [7, 8]. Autochthonous malaria cases have numbered in only the hundreds annually during the past several years [7–10]. The call for global malaria elimination advocated by WHO was responded actively in P.R.China, with a national malaria elimination action plan being launched by the Chinese central government in 2010, which intends to reach the goal of malaria elimination nationwide by 2020 [11–13]. A challenge is the globalization strategy for economic development in P.R.China, which is resulting in more cases of imported malaria in recent years [14–16]. Of particular concern is the threat to individual health of Chinese citizens travelling abroad, and the potential re-introduction of local transmission in malaria-free areas when travellers return. To facilitate better response strategies for this new challenge, the epidemiological features of imported malaria need to be further explored. In this study, a case-based epidemiological survey on each imported malaria case was conducted in nine provinces of China. The characteristics of importation origin, *Plasmodium* species, prevention, infection, and clinical outcome of imported malaria are described.

## Methods

In P.R.China, malaria cases are diagnosed by clinicians in accordance with the unified national diagnostic criteria. Laboratory-confirmed malaria cases refer to patients with any positive result in the following diagnostic tests relating to malaria: malaria parasites confirmed by microscopy, rapid diagnostic tests (RDT), or polymerase chain reaction (PCR) tests. Since the initiation of the National Malaria Elimination Action Plan in 2010, each case should be investigated by local staff at county level of the Centre of Disease Control and Prevention, and each case should be classified as local or imported malaria [11]. A malaria patient is classified as an imported case if the individual travelled to a malaria-endemic country within the previous month [17]. In this study, the last country destination of travel was taken as the origin of malaria infection; a person who went abroad as a member of a group organized by an agency was identified as a group traveller; otherwise a person was identified as an individual traveller.

All imported malaria cases reported in the National Infectious Disease Reporting Information System in nine selected provinces of China were further investigated on their detailed travel history, preventive measure, clinical presentation and outcome. Among all 31 provinces in mainland China, nine provinces were selected as the study setting, based on a willingness to participant in the survey, geographic location, levels of incidence of malaria in the past 3 years (2010–2012), population, and numbers of overseas travellers in 2012 (Table 1) [18, 19]. The overall population in the nine selected provinces was 0.58 billion, which accounted for 43.3 % of the population in the whole country. During the period of 1 November, 2013 to 30 October, 2014, each imported laboratory-confirmed malaria case in these nine provinces was interviewed face-to-face, using a standard questionnaire, by the epidemiological staff at the county level of the Centre for Disease Control and Prevention. Individual-level information on age, gender, residence address, medical care-seeking process, symptoms, laboratory test results, travel history, clinical presentation, and outcome were collected. As one part of national malaria elimination plan, this study was approved by the Chinese Centre for Disease Control and Prevention, and all individual identification information was concealed in the database when performing data analysis.

**Table 1:** Background information on the nine study provinces for imported malaria surveys, in China

Areas	Geographic location	Population in 2012 (000s)	International travellers in 2012^a^ (000s)	Total malaria cases 2010–2012
Entire country	–	1,347,890	116,265.7	13,928
Overall of nine study provinces (% of entire country)	–	584,060 (43.3 %)	61,944.0 (53.3 %)	7296 (52.4 %)
Liaoning	Northeast	43,890	4731.3	38
Gansu	Northwest	25,780	102.0	278
Henan	Central	94,060	1907.7	1358
Shandong	Central	96,850	4699.1	479
Shanghai	East	23,800	6512.3	117
Hunan	South	66,390	2245.5	323
Guangdong	South	105,940	34,894.3	91
Sichuan	Southwest	80,760	2273.4	667
Yunnan	Southwest	46,590	4578.4	3945

^a^Refers to person-times of overseas visitors who enter mainland China

Demographic data, *Plasmodium* species profiles, spatiotemporal distribution of cases, case detection and clinical features, as well as preventive measures and exposure histories during travel abroad were analysed. A seasonal index was used to understand the seasonal patterns of imported malaria occurrence. An index for a given month (i.e., May) was calculated by case numbers for that month (i.e., May) divided by the monthly mean of cases during the whole 12 months of the survey [20]. No obvious seasonal pattern was expected if the seasonal index of each month was close to 1.0. Medical service providers for malaria diagnosis were categorized by private clinic, primary hospital, hospital at county level, and hospital at city level, according to their population coverage and the techniques, equipment, and staff available.

## Results

### Demographic profile

From 1 Nov, 2013 to 30 Oct, 2014, a total of 1420 imported malaria cases were reported in the study provinces. The mean age of the imported malaria cases was 36.8 years old (range 1–69, IQR 28–45), and 87.4 % (1241 cases) were aged between 21 and 50 years. Males accounted for 95 % of all cases. Overseas labourers were the most frequent occupational group with malaria infection, accounting for 81.8 % of all imported cases.

### *Plasmodium* species by origin and location

*P. falciparum* (723 cases, 50.9 %) and *P. vivax* (629 cases, 44.3 %) were the two predominant species. Only 26 *P. malariae* cases and 31 *P. ovale* cases were detected. Nine mixed infections were recorded, which included seven cases of *P. falciparum* mixed with *P. vivax*, one case of *P. falciparum* and *P. malariae*, and one case of *P. vivax* and *P. malariae*. The remaining two malaria cases were not sub-typed by species.

**Fig. 1:**
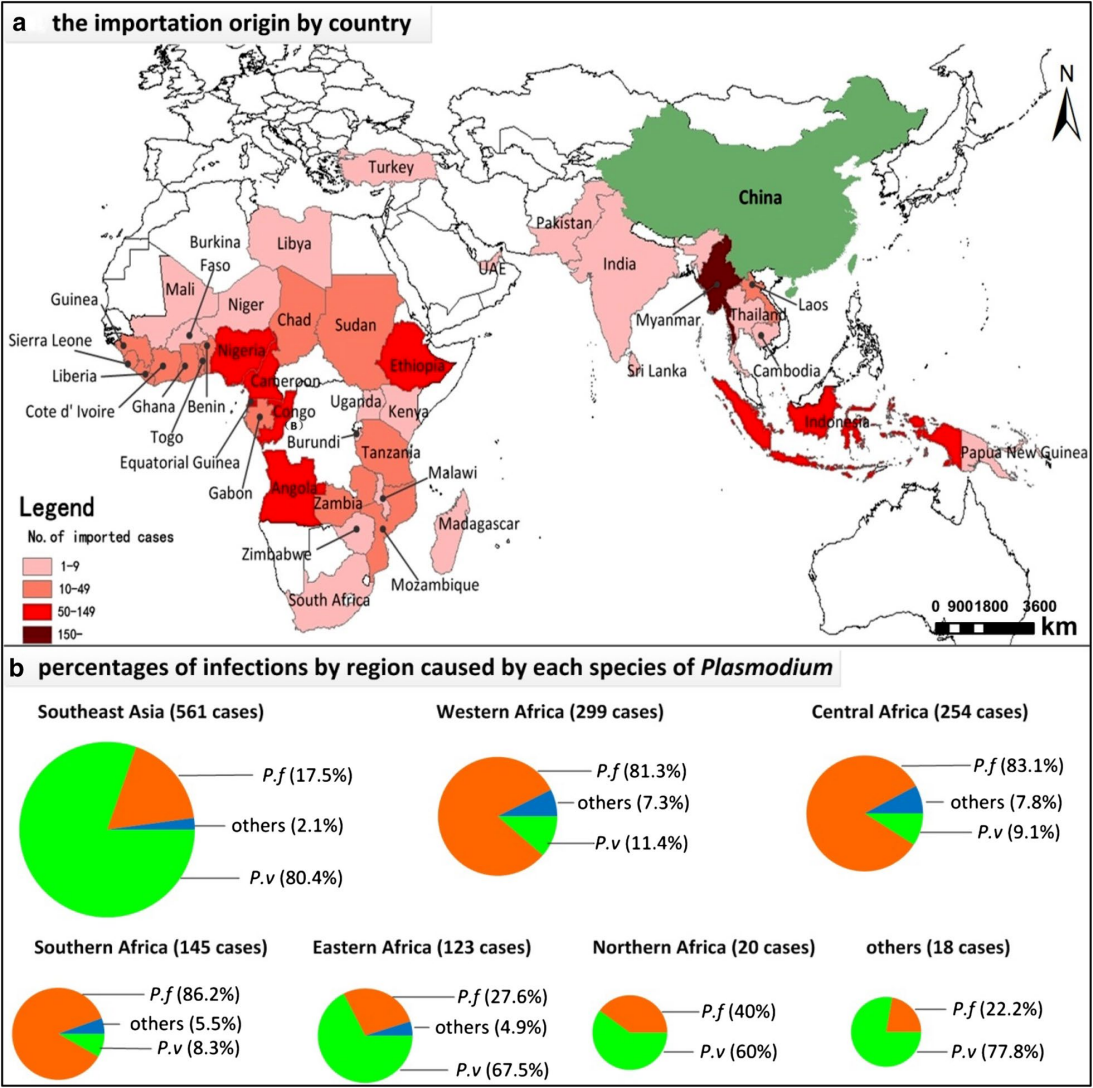
The geographic location of importation origin by country and the *Plasmodium* species of imported malaria by region. (**a** the importation origin by country, **b** percentages of infections by region caused by each species of *Plasmodium*, *P. f Plasmodium falciparum, P. v Plasmodium vivax,* others including *Plasmodium malariae* and *Plasmodium ovale*)

Imported cases came from 41 countries located in Africa, Asia, Oceania, and Europe, with Africa (841 cases, 59.2 %) and Southeast Asia (561 cases, 39.5 %) being the most common regions of origin. Myanmar was by far the leading country with 452 imported cases (Fig. 1a).

The major species of imported malaria varied by sub-region. *P. vivax* predominated in imported cases returning from Southeast Asia (80.4 %) and eastern Africa (67.5 %), while *P. falciparum* predominated in cases from western Africa (81.3 %), central Africa (83.1 %), and southern Africa (86.2 %). *P. malariae* and *P. ovale* mainly came from central and West Africa (Fig. 1b).

Among the 1094 counties of the nine study provinces, 25.5 % (279 counties) were affected by imported malaria cases. The three leading provinces in terms of numbers of cases were Yunnan (477 cases), Sichuan (249 cases) and Henan provinces (201 cases). In Yunnan Province, three counties that shared land borders with Myanmar were the most severely affected with more than 50 imported cases each. The majority of imported cases (933 cases, 65.7 %) came from non-adjacent countries to China, and the remaining 487 cases (34.3 %) had returned from adjacent countries (Myanmar, Laos, India, and Pakistan).

**Fig. 2:**
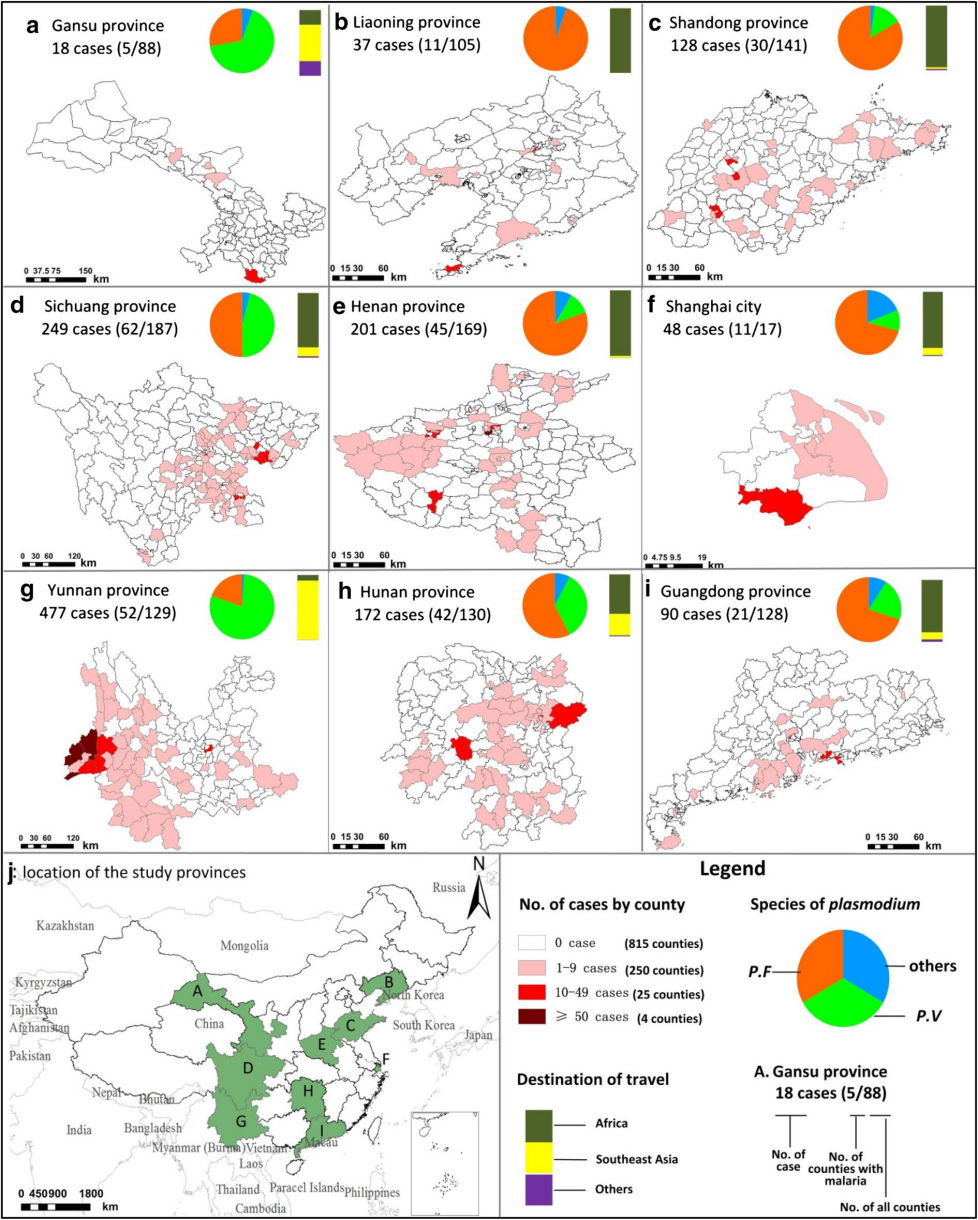
The spatial distribution of imported malaria by county, the species of *Plasmodium* and travel destination of the imported malaria by province in the nine study provinces of China. **a** Gansu province, **b** Liaoning province, **c** Shandong province, **d** Sichuan province, **e** Henan province, **f** Shanghai city, **g** Yunnan province, **h** Hunan province, **i** Guangdong province, **j** Location of the study nine provinces

The main species of *Plasmodium* varied among the study provinces. In Yunnan and Gansu provinces, most of the cases (89.7 %, 444/495) came from Southeast Asia, with *P. vivax* being the most predominant species. Most of the imported cases (85.5 %, 791/925) in the other seven provinces, including Liaoning, Shandong, Sichuan, Henan, Shanghai, Hunan, and Guangdong, had returned from Africa, with the predominant species being *P. falciparum* (Fig. 2).

### Temporal features

**Fig. 3:**
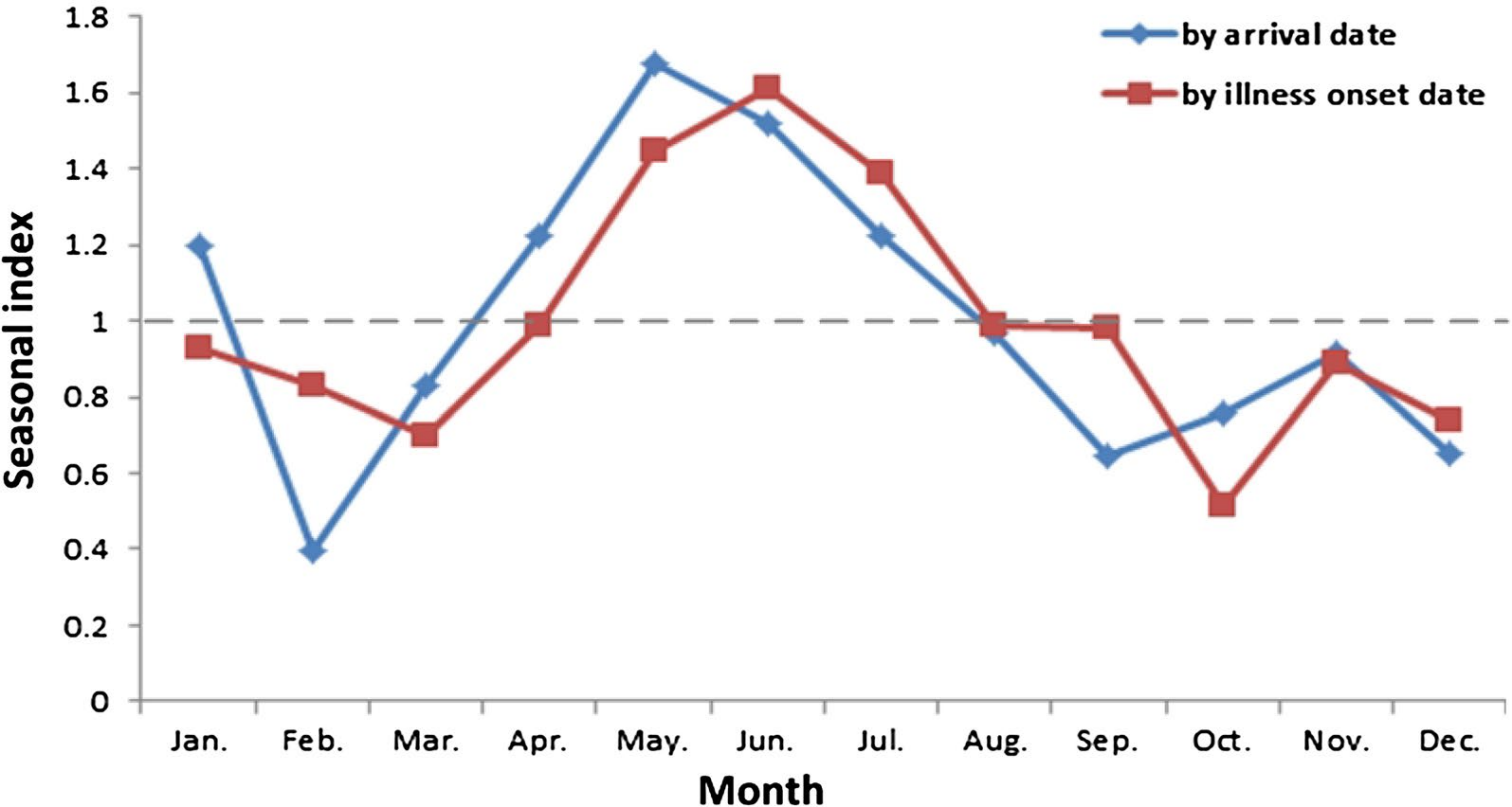
The monthly distribution of imported malaria cases by arrival date and illness onset date in the study provinces of China, 2013–2014

There were on average 118 imported malaria cases per month (range: 61–191), with May (198 cases) and June (179 cases) having the highest numbers according to arrival date, with a seasonal index of 1.7 and 1.5 in May and June, respectively (Fig. 3). Similarly, May, June and July had the highest numbers of cases by disease onset date. The median interval between arrival date in China and onset of illness was 8 days (IQR 2.8–21 days), with 84 % of the cases developing the illness within 30 days after arrival, and 91.8 % within 60 days.

### Case detection and clinical outcome

More than 22 % of imported cases sought medical assistance through private clinics and primary hospitals after having developed the illness. Imported malaria cases were laboratory diagnosed after having experienced an average of 1.1 medical visits, with 107 cases being confirmed by laboratory testing after two or more visits. In total, 78.8 % of all cases were laboratory confirmed by hospitals at county level and above. During the initial medical care visit, the overall proportion of malaria testing performed was 73.4 % among all levels of medical institution, with only 7.9 % of the total in private clinics and 30.2 % in primary hospitals (Table 2).

**Table 2:** The detection of imported malaria cases by different health providers in nine provinces of China

Item	Overall	Private clinics	Primary hospitals	Hospitals at county level	Hospitals at city level
Number (%) of initial medical visit	1420	215 (15.1)	106 (7.5)	443 (31.2)	656 (46.2)
Proportion of cases for whom laboratory testing was done during the initial medical visit % (no. tested/all)	73.4 (1042/1420)	7.9 (17/215)	30.2 (32/106)	87.1 (386/443)	87.7 (575/656)
Number (%) of laboratory-confirmed cases	1420	153 (10.8)	148 (10.4)	406 (28.6)	713 (50.2)
Proportion of cases for whom a travel history was recorded during the initial visit % (no. recorded/all)	82.7 (1175/1420)	37.7 (81/215)	80.2 (85/106)	78.1 (346/443)	95.6 (627/656)

Proportion of cases for whom a travel history to malaria-endemic countries was recorded during the initial visit was found to be as high as 95.6 % in patients attending hospitals at city level, but was much lower in private clinic (37.7 %). The median intervals were 2 days (IQR 0–4 days) from symptom onset to first medical visit and 2 days (IQR 0–5 days) from the first medical visit to malaria diagnosis.

Among the 1420 imported cases, 945 were treated in an outpatient department, and 475 cases were hospitalized for treatment (with a hospitalized proportion of 33.4 %), of whom 12 died (giving a case fatality rate of 0.8 %). The four leading symptoms were fever (97.9 %), chills (81.6 %), sweating (71.0 %), and headache (64.9 %) (Table 3). A total of 112 cases (7.9 %) developed complicated symptoms, the most frequent among in-patients being liver function impairment (8.0 %), gastrointestinal damage (6.7 %), acute renal dysfunction (5.3 %), and coma (5.3 %). The proportion of hospitalization for *P. falciparum* cases was 46.2 % (334/723), which was much higher than that for *P. vivax* cases (18.1 %, 114/629). Among the 12 deaths, the median age was 46 years (range 31–55), which was significantly higher than that of the survivors (37 years, range 1–69) (two independent samples Wilcoxon test, Z = −2.36, *p* = 0.018).

**Table 3:** Clinical manifestation of imported malaria cases by outpatient and inpatient in nine provinces of China

Clinical features	Overall (n = 1420)	Outpatients n = 945			Inpatients n = 475		
		*P.f.* (n = 389)	*P.v.* (n = 515)	All (n = 945)	*P.f.* (n = 334)	*P.v.* (n = 114)	All (n = 475)
Common signs/symptoms	1412 (99)	383 (98.5 %)	514 (99.8 %)	938 (99.3 %)	333 (99.7 %)	113 (99.1 %)	474 (99.8 %)
Fever	1390 (97.9 %)	372 (95.6 %)	509 (98.8 %)	920 (97.4 %)	331 (99.1 %)	113 (99.1 %)	470 (98.9 %)
Chills	1159 (81.6 %)	282 (72.5 %)	474 (92.0 %)	785 (83.1 %)	248 (74.3 %)	104 (91.2 %)	374 (78.7 %)
Sweating	1008 (71.0 %)	249 (64.0 %)	423 (82.1 %)	697 (73.8 %)	203 (60.8 %)	90 (78.9 %)	311 (65.5 %)
Headache	921 (64.9 %)	234 (60.2 %)	383 (74.4 %)	640 (67.7 %)	188 (56.3 %)	79 (69.3 %)	281 (59.2 %)
Malaise	414 (29.2 %)	122 (31.4 %)	147 (28.5 %)	281 (29.7 %)	92 (27.5 %)	35 (30.7 %)	133 (28.0 %)
Dizziness	272 (19.2 %)	89 (22.9 %)	85 (16.5 %)	181 (19.2 %)	66 (19.8 %)	22 (19.3 %)	91 (19.2 %)
Diarrhoea	168 (11.8 %)	42 (10.8 %)	41 (8.0 %)	87 (9.2 %)	44 (13.2 %)	5 (4.4 %)	81 (17.1 %)
Complicated signs/symptoms	112 (7.9 %)	0	0	0	95 (28.4 %)	17 (14.9 %)	112 (23.6 %)
Coma	38 (2.7 %)	0	0	0	25 (7.5 %)	0	25 (5.3 %)
Cerebral lesion	32 (2.3 %)	0	0	0	22 (6.6 %)	1 (0.9 %)	23 (4.8 %)
Gastro-intestinal damage	25 (1.8 %)	0	0	0	23 (6.9 %)	9 (7.9 %)	32 (6.7 %)
Liver function impairment	25 (1.8 %)	0	0	0	38 (11.4 %)	0	38 (8.0 %)
Acute renal dysfunction	23 (1.6 %)	0	0	0	23 (6.9 %)	0	23 (4.8 %)
Severe anaemia	23 (1.6 %)	0	0	0	21 (6.3 %)	4 (3.5 %)	25 (5.3 %)
Haemolysis	19 (1.3 %)	0	0	0	17 (5.1 %)	2 (1.8 %)	19 (4.0 %)
Shock	7 (0.5 %)	0	0	0	7 (2.1 %)	0	7 (1.5 %)
Acidosis	5 (0.4 %)	0	0	0	4 (1.2 %)	1 (0.9 %)	5 (1.1 %)

*P.f. Plasmodium falciparum, P.v.* *Plasmodium vivax*

### Preventive measures and infection history

Among the 1261 cases (88.8 % of total) who travelled individually or as a part of a group was determined, 738 cases (58.5 %) went abroad as a group, and 523 cases (41.5 %) travelled individually. Of the group travellers, 52.4 % had been trained on malaria prevention measures by the organizing agency. Anti-malarial medication was obtained by 27.8 % of the cases prior to their overseas travel; the figure was 11.3 % among individual travellers. Mosquito repellents were obtained by 40.5 % of patients before travel; the figure was 21.4 % for individual travellers. The overall proportion of bed net usage during the period abroad was 73.4 %. The median period abroad was 157 days; for group travellers, the period was longer (221 days) than for individual travellers (72 days). Nearly half of the cases (50.4 %) had been diagnosed with malaria infection during the period abroad and 27.7 % of the cases had experienced two or more episodes of malaria infection.

## Discussion

This study found that a large number of imported malaria cases were detected in P.R.China, and that overseas labourers were the most frequently affected group. The countries of origin of the infections were widely distributed in Africa and Southeast Asia. Many imported cases presented with complicated symptoms, leading to 12 deaths. The awareness of clinical manifestations and the capacity for malaria diagnosis were weak in private clinics and primary healthcare facilities.

Due to global economic integration and the rapid economic development of China, large numbers of Chinese people travel to malaria-endemic countries for financial investment, commercial trade, labour, and tourism. According to the report from the Chinese Bureau of Exit and Entry Administration, more than 83 million people went abroad to seek job opportunities, travel or study overseas in 2012 [21]. The relationship between the increased economic investment and numbers of exported labourers to Africa from China and the increased number of cases imported has been well established [15]. Overseas labourers engaged in road or bridge building, mining and other outdoor activities are the highest risk group for malaria infection [14–16]. Overseas labourers usually work on construction sites and experience poor living conditions with a lack of access to mosquito control measures. Additionally, Chinese labourers generally lack immunity to local *Plasmodium* species, especially to *P.falciparum* in Africa, and exported labourers are generally poorly educated and lack awareness of the risk of malaria and personal protection against mosquito bites [22]. Guidelines on malaria chemoprophylaxis for international travellers should be developed in China, so as to reduce the risk of malaria infection among high-risk groups.

With the widespread occurrence of imported malaria, several threats are now facing China. Firstly, a large number of imported cases were due to *P. falciparum*, the species most commonly associated with severe disease and death, meaning that prompt diagnosis and appropriate treatment are critical [3]. However, this *Plasmodium* species was relatively rare in most settings in China, and most healthcare workers at primary level lack awareness and skills to diagnose and manage cases infected with *falciparum* malaria [23]. Furthermore, a report on one large-scale cluster of imported malaria cases returning from Ghana showed that about 34.4 % of *Plasmodium*-positive persons had asymptomatic infections [14], which further complicates timely detection by routine malaria surveillance system. Therefore, it is estimated in this study that nearly 30 % of imported cases were likely under-reported. In addition, whilst most local transmission of malaria in China has been successfully interrupted [24], wide distribution of *A.sinensis* throughout the country may make many areas receptive to transmission [25]. As a result, imported *vivax* malaria may lead to re-introduction in areas that have been free of malaria for many years [26, 27], presenting a threat to nationwide malaria elimination by 2020.

Along with the rapid development of international trade and overseas travel in China, it is expected that the situation of disease importation will become more problematic if effective preventive measures are not undertaken [9, 28]. Malaria infection prevention measures, intensive surveillance and medical service delivery to exported labourers should be prioritized by the Chinese public health authorities [13, 29]. Intersectorial cooperation between public health, medical, commercial, and travel sectors could play a critical role in the prevention, detection and management of imported malaria. Training of local epidemiologists and physicians on malaria case diagnosis and investigation needs to be enhanced. The epidemiological features of imported malaria cases and the impact of imported cases on malaria elimination in China should be further explored with long-term data.

One of the limitations on this study was that, as the survey was retrospectively performed, the information on imported case exposure, infection and treatment history when staying abroad may have some recall bias, given that the travellers had lived and worked overseas for a long time at the time of survey. In addition, as only imported cases diagnosed as malaria were enrolled in this study, the population of travellers returned to China from various countries during the survey period was unavailable, and thereby the incidence of malaria among overseas travellers could not be estimated.

## Conclusions

This study shows that overseas infections of malaria have become a major threat to Chinese labourers travelling to countries in West Africa, East Africa, and Southeast Asia. In order to reduce the infection risk of malaria during periods abroad, awareness and effective protective measures against exposure to mosquitoes and malaria parasites among high-risk groups should be enhanced. The need to improve capacity for imported case detection and the timeliness of anti-malarial treatments should be highlighted, so as to reduce burden of severe malaria disease and deaths, as well as prevent secondary malaria transmission within China.
