# Erratum to: Epidemiologic features of overseas imported malaria in the People’s Republic of China

**DOI:** 10.1186/s12936-016-1355-x

**Published:** 2016-06-14

**Authors:** Zhongjie Li, Qian Zhang, Canjun Zheng, Sheng Zhou, Junling Sun, Zike Zhang, Qibin Geng, Honglong Zhang, Liping Wang, Shengjie Lai, Wenbiao Hu, Archie C. A. Clements, Xiao-Nong Zhou, Weizhong Yang

**Affiliations:** Division of Infectious Diseases, Key Laboratory of Surveillance and Early-Warning on Infectious Disease, Chinese Center for Disease Control and Prevention, 155 Changbai Road, Changping, 102206 Beijing, China; Center of Clinical Laboratory, The First Affiliated Hospital, College of Medicine, Zhejiang University, Hangzhou, China; State Key Laboratory of Virology and College of Life Sciences, Wuhan University, Wuhan, China; Department of Geography and Environment, University of Southampton, Southampton, UK; School of Public Health and Social Work, Queensland University of Technology, Brisbane, Australia; Research School of Population Health, College of Medicine, Biology and Environment, The Australian National University, Canberra, Australia; National Institute of Parasitic Diseases, Chinese Center for Disease Control and Prevention, Key Laboratory of Parasite and Vector Biology, MOH, WHO Collaborating Centre for Tropic Diseases, National Center for International Research on Tropical Diseases, 207 Rui Jin Er Road, Shanghai, 200025 People’s Republic of China; Key Laboratory of Surveillance and Early-Warning on Infectious Disease, Chinese Center for Disease Control and Prevention, Beijing, China

## Erratum to: Malar J (2016) 15:141 DOI 10.1186/s12936-016-1188-7

The authors of [1] would like to highlight the following corrections to their original article published in *Malaria Journal*.

In the third paragraph of ‘Case detection and clinical outcome’ in the “Results” section of [1], “acute renal dysfunction (5.3 %)” should be replaced as “severe anaemia (5.3 %)”.

In addition, in Table 3 of the original publication [1], the Overall Complicated signs/symptoms on coma should be 25 (1.8 %), Cerebral lesion be 23 (1.6 %), Gastrointestinal damage 32 (2.3 %), Liver function impairment 38 (2.7 %) and Severe anaemia 25 (1.8 %). The following is our corrected Table 3.

**Table 3:** Clinical manifestation of imported malaria cases by outpatient and inpatient in nine provinces of China

Clinical features	Overall (n = 1420)	Outpatients n = 945			Inpatients n = 475		
		*P.f.* (n = 389)	*P.v.* (n = 515)	All (n = 945)	*P.f.* (n = 334)	*P.v.* (n = 114)	All (n = 475)
Common signs/symptoms	1412 (99)	383 (98.5 %)	514 (99.8 %)	938 (99.3 %)	333 (99.7 %)	113 (99.1 %)	474 (99.8 %)
Fever	1390 (97.9 %)	372 (95.6 %)	509 (98.8 %)	920 (97.4 %)	331 (99.1 %)	113 (99.1 %)	470 (98.9 %)
Chills	1159 (81.6 %)	282 (72.5 %)	474 (92.0 %)	785 (83.1 %)	248 (74.3 %)	104 (91.2 %)	374 (78.7 %)
Sweating	1008 (71.0 %)	249 (64.0 %)	423 (82.1 %)	697 (73.8 %)	203 (60.8 %)	90 (78.9 %)	311 (65.5 %)
Headache	921 (64.9 %)	234 (60.2 %)	383 (74.4 %)	640 (67.7 %)	188 (56.3 %)	79 (69.3 %)	281 (59.2 %)
Malaise	414 (29.2 %)	122 (31.4 %)	147 (28.5 %)	281 (29.7 %)	92 (27.5 %)	35 (30.7 %)	133 (28.0 %)
Dizziness	272 (19.2 %)	89 (22.9 %)	85 (16.5 %)	181 (19.2 %)	66 (19.8 %)	22 (19.3 %)	91 (19.2 %)
Diarrhoea	168 (11.8 %)	42 (10.8 %)	41 (8.0 %)	87 (9.2 %)	44 (13.2 %)	5 (4.4 %)	81 (17.1 %)
Complicated signs/symptoms	112 (7.9 %)	0	0	0	95 (28.4 %)	17 (14.9 %)	112 (23.6 %)
Coma	25 (1.8 %)	0	0	0	25 (7.5 %)	0	25 (5.3 %)
Cerebral lesion	23 (1.6 %)	0	0	0	22 (6.6 %)	1 (0.9 %)	23 (4.8 %)
Gastro-intestinal damage	32 (2.3 %)	0	0	0	23 (6.9 %)	9 (7.9 %)	32 (6.7 %)
Liver function impairment	38 (2.7 %)	0	0	0	38 (11.4 %)	0	38 (8.0 %)
Acute renal dysfunction	23 (1.6 %)	0	0	0	23 (6.9 %)	0	23 (4.8 %)
Severe anaemia	25 (1.8 %)	0	0	0	21 (6.3 %)	4 (3.5 %)	25 (5.3 %)
Haemolysis	19 (1.3 %)	0	0	0	17 (5.1 %)	2 (1.8 %)	19 (4.0 %)
Shock	7 (0.5 %)	0	0	0	7 (2.1 %)	0	7 (1.5 %)
Acidosis	5 (0.4 %)	0	0	0	4 (1.2 %)	1 (0.9 %)	5 (1.1 %)

P.f., *Plasmodium falciparum*; P.v., *Plasmodium vivax*

We regret these mistakes.

## References

[1] Li Z, Zhang Q, Zheng C, Zhou S, Sun J, Zhang Z (2016). Epidemiologic features of overseas imported malaria in the People’s Republic of China. Malar J.

